# A wide spectrum of phenotype of deficiency of deaminase 2 (DADA2): a systematic literature review

**DOI:** 10.1186/s13023-023-02721-6

**Published:** 2023-05-13

**Authors:** Ilaria Maccora, Valerio Maniscalco, Silvia Campani, Simona Carrera, Giulia Abbati, Edoardo Marrani, Maria Vincenza Mastrolia, Gabriele Simonini

**Affiliations:** 1 Rheumatology Unit, ERN ReConnet Center, Meyer Children’s Hospital IRCCS, Florence, Italy; 2 NeuroFARBA Department, University of Florence, Florence, Italy; 3 School of Health Science, University of Florence, Florence, Italy

**Keywords:** DADA2, Deficiency of adenosine deaminase 2, Systematic review, Autoinflammatory disease, Humoral immunodeficiency, Vasculitis, Stroke, Panarteritis nodosa

## Abstract

**Introduction:**

Deficiency of adenosine deaminase 2 (DADA2) is a rare monogenic autoinflammatory disease, whose clinical phenotype was expanded since the first cases, originally described as mimicker of polyarteritis nodosa, with immunodeficiency and early-onset stroke.

**Methods:**

A systematic review according to PRISMA approach, including all articles published before the 31st of August 2021 in Pubmed and EMBASE database was performed.

**Results:**

The search identified 90 publications describing 378 unique patients (55.8% male). To date 95unique mutations have been reported. The mean age at disease onset was 92.15 months (range 0–720 months), 32 (8.5%) showed an onset of the first signs/symptoms after 18 years old and 96 (25.4%) after 10 years old. The most frequent clinical characteristics described were cutaneous (67.9%), haematological manifestations (56.3%), recurrent fever (51.3%), neurological as stroke and polyneuropathy (51%), immunological abnormalities (42.3%), arthralgia/arthritis (35.4%), splenomegaly (30.6%), abdominal involvement (29.8%), hepatomegaly (23.5%), recurrent infections (18.5%), myalgia (17.9%), kidney involvement (17.7%) etc. Patients with skin manifestations were older than the others (101.1 months SD ± 116.5, vs. 75.3 SD ± 88.2, p 0.041), while those with a haematological involvement (64.1 months SD ± 75.6 vs. 133.1 SD ± 133.1, p < 0.001) and immunological involvement (73.03 months SD ± 96.9 vs. 103.2 SD ± 112.9, p 0.05) are younger than the others. We observed different correlations among the different clinical manifestations. The use of anti-TNFα and hematopoietic cell stems transplantation (HCST) has improved the current history of the disease.

**Conclusion:**

Due to this highly variable phenotype and age of presentation, patients with DADA2 may present to several type of specialists. Given the important morbidity and mortality, early diagnosis and treatment are mandatory.

**Supplementary Information:**

The online version contains supplementary material available at 10.1186/s13023-023-02721-6.

## Background

Deficiency of adenosine deaminase 2 (DADA2) is a rare monogenic autoinflammatory disease that affects several organs and systems with mutable clinical manifestations. After the first cases described in 2014 by two independent groups as a mimicker of polyarteritis nodosa (PAN), of note associated with immunodeficiency, and early-onset stroke [1, 2], the clinical phenotype was expanded [3–5].

An homozygous or compound heterozygous mutation causing a loss-of-functions in the gene of the Adenosine Deaminase 2 (ADA2), which encodes the homonymous enzyme, is the cause of this rare syndrome [1, 2].

Conversely, the pathogenesis is less clear. The enzymatic deficiency leads to a switch in myeloid cells toward a prevalent pro-inflammatory M1 macrophages with an increased release of proinflammatory cytokines. Tumor necrosis factor-alpha (TNF-α) seems to have a key role in the pathogenesis of the disease. TNF-α induces a chronic upregulation of the neutrophil activity and an endothelial cell instability that eventually prone to a vasculitis phenotype and bone marrow disorders [1, 2].

Currently, TNF-α inhibitors are the treatment of choice in DADA-2 patients and results effective on controlling the vasculitis signs and preventing the strokes [6]. In patients with humoral immunodeficiency and recurrent infections, immunoglobulin replacement (intravenously or subcutaneously) has been used [1, 7–12]. Hematopoietic stem cell transplantation (HSCT) has been attempted as rescue therapy, especially in patients with severe hematologic and immunologic disorders. This treatment is however burdened by well-known side effects [4, 12, 13].

Over the time the clinic and therapeutic knowledge of the disease advanced ameliorating the patient’s management and prognosis. To date over 300 cases have been described, however mainly as single case reports or small cohort of patients.

Herein, in a systematic literature review we aimed to summarize the current evidence about the disease manifestations and available treatments of DADA2.

## Main text

### Materials and methods

A systematic review was conducted following the Preferred Reporting Items for Systematic Reviews and Meta-Analyses (PRISMA) statement and the protocol followed the PRISMA guidelines [14].

### Search strategy

We searched in the following electronic bibliographic databases MEDLINE via PubMed and EMBASE up to the 31st of August 2021. The search was performed in all fields using the following keywords (“Deficiency of Adenosine Deaminase 2” OR “DADA2” OR “ADA2 deficiency” OR “deficit of adenosine deaminase 2” OR “CECR1”).

Language restriction was applied to evaluate only papers written in English.

This review included registries, retrospective cohort, prospective cohort studies, case series and case reports. Experimental and quasi experimental studies including clinical trials and open label studies may report data on new case of DADA2 have been considered. Animal studies, conference abstract and review articles have been excluded.

Title and abstract screening were performed independently by two reviewers, at least (IM, SC, SC, VM). Full-text screening was performed by two different independent couples of reviewers (IM and SC, VM and SC). When there was a disagreement, it was resolved through discussion with a third reviewer (GS).

### Eligibility criteria

To be included in this review papers need to report: (a) data about paediatric and adult patients with DADA2 syndrome confirmed with the genetic analysis; (b) description of the clinical phenotype of the patients.

Paper not reporting the following information have been excluded: (a) non confirmed diagnosis of DADA2 syndrome; (b) no data about the clinical phenotype.

### Outcome measures

The main outcome measure is the description of the clinical phenotype of patients with DADA2 and different therapies performed.

### Data extraction

Data have been extracted independently by 2 reviewers. Any disagreements have been solved through consensus between the 2 reviewers. Based on the anticipated quantitative data to be extracted not many disagreements were anticipated. However, if unresolved disagreement occurred, an arbitrator (GS) was used. Data extraction included first author, title of study, year of publication, type of study, mutation (in terms of homozygous, heterozygous, compound heterozygous, specific mutation showed by the patients, specific site of the mutation), demographics characteristics (sex distribution, ethnicity, age at onset of symptoms), clinical phenotype (recurrent fever, growth retardation, cutaneous manifestation (PAN; aspecific rash…), haematological manifestation (bone marrow depression, anaemia, thrombocytopenia, lymphopenia), gastrointestinal manifestation, cardiac manifestation, CNS manifestations, immunological manifestation (hypogammaglobulinemia, B cell deficiency, T cell deficiency, combined deficiency others), ocular manifestation, gastrointestinal manifestation mutation. Response to each therapy performed.

To avoid reporting the same patient multiple times (duplicated case selection bias), we followed the specific advises of the PRISMA guidelines: during the data extraction phase, the reviewers in charge do not further included patients when the authors specified, according to data reporting guidelines, that index patients were previously reported in other papers. When the patient was a duplicated, the most updated and complete information was considered. When we have been unable to ascertain if the index patient was a duplicate, we directly mailed to the authors. If no reply obtained, we decided case by case in meeting discussion. If any doubt, we adopted a conservative method excluding the index patient.

Data have been collected and organized using Microsoft Excel.

### Statistical analysis

Statistical analysis has been performed with SPSS 27.0. Due to the heterogeneity of studies regarding study designs, participants, measures and outcomes, a descriptive systematic literature review has been conducted. We reported continuous variables as mean and standard deviation, and as number (percentages) for categorical variables. We used the chi-square and Fisher’s exact test as appropriate to compare qualitative variable. Pearson and Spearman correlation tests, properly adjusted for multiple comparisons, were used to determine correlation coefficients for different variables. Nonparametric tests were used, where necessary, due to the small size of the groups and to the skewness of our data. A p-value < 0.05 was considered statistically significant.

## Results

The literature search produced 1420 records (Embase: 1005, PubMED: 415). After deduplication, 1123 papers have been screened for title and abstract evaluation and 116 papers have been included for full text evaluation. One thousand and seven papers were excluded based on the title and abstract evaluation because 380 were not eligible for publication type, 236 for wrong outcomes and 391 for population not eligible.

After full text screening 90 papers have been used in our systematic review [1–13, 15–91]: 8 papers were excluded for the publication type and 18 because they did not report the clinical phenotype or the confirmatory genetic mutations.

**Fig. 1:**
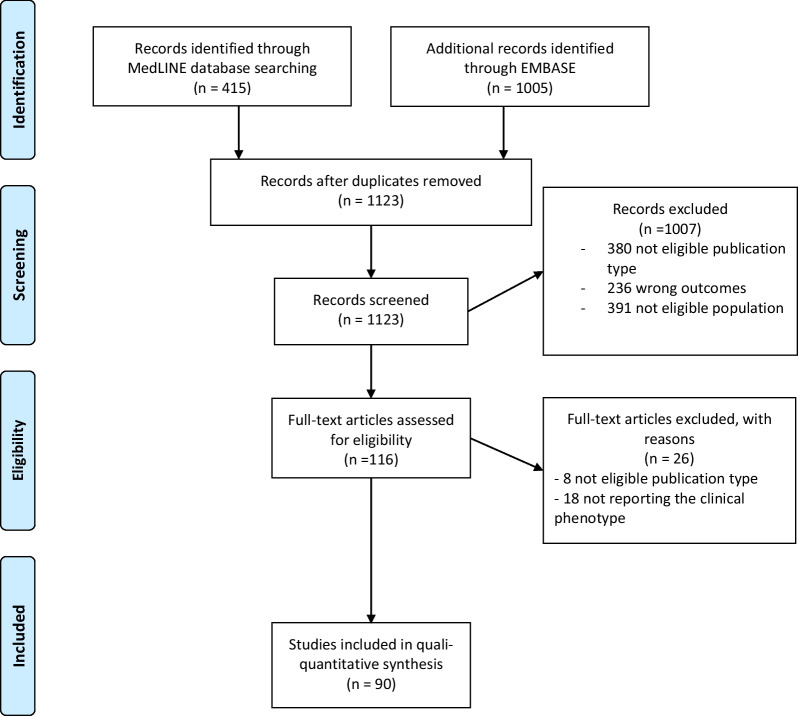
PRISMA flow diagram. From: Moher D, Liberati A, Tetzlaff J, Altman DG, The PRISMA Group (2009). Preferred Reporting Items for Systematic Reviews and Meta-Analyses: The PRISMA Statement. PLoS Med 6(7): e1000097. DOI: 10.1371/journal.pmed1000097

Figure 1 represented the PRISMA flow diagram demonstrating the process of study selection.

### Genetic characteristics of the populations

Ninety-five unique pathogenetic/likely pathogenetic mutations have been reported to date (see Additional file 1: Table S1). Two-hundred and fifteen patients showed a homozygous mutation, 122 showed a compound heterozygosity (biallelic mutation with two different mutations) and 2 a heterozygosity for a single mutation. In 39 patients the mutations reported are not described in the papers, but they specified that patients had a genetically confirmed diagnosis. Among the 329 eligible patients, information regarding the ADA2 enzymatic activity was available only for 175 subjects. In 174/175 patients (99.5%), including the 2 with a single mutation in heterozygosis [62], a decreased activity of Adenosine Deaminase 2 was reported. In one patient, with confirmed homozygous mutation, the ADA2 activity that resulted at normal ranges, was detected after bone marrow transplantation [10].

### Clinical characteristics

Three-hundred and seventy-eight patients have been identified, of whom 211 male (55.8%), with a mean age at disease onset of 92.15 months (SD ± 108.2, range 0–720 months), at immunodeficiency onset of 85.4 months (SD ± 114, range 3–480 months) and at first stroke 82.9 (SD ± 139.4, range 5–768 months). Among the 378 patients, 32 (8.5%) showed an onset of the first signs/symptoms after 18 years old and 96 (25.4%) after 10 years old. The spectrum of manifestations at disease onset were widely variables including recurrent fever, cutaneous involvement as panarteritis nodosa-like, recurrent infections, haematological findings, strokes or immunodeficiency or polyneuropathies.

**Fig. 2:**
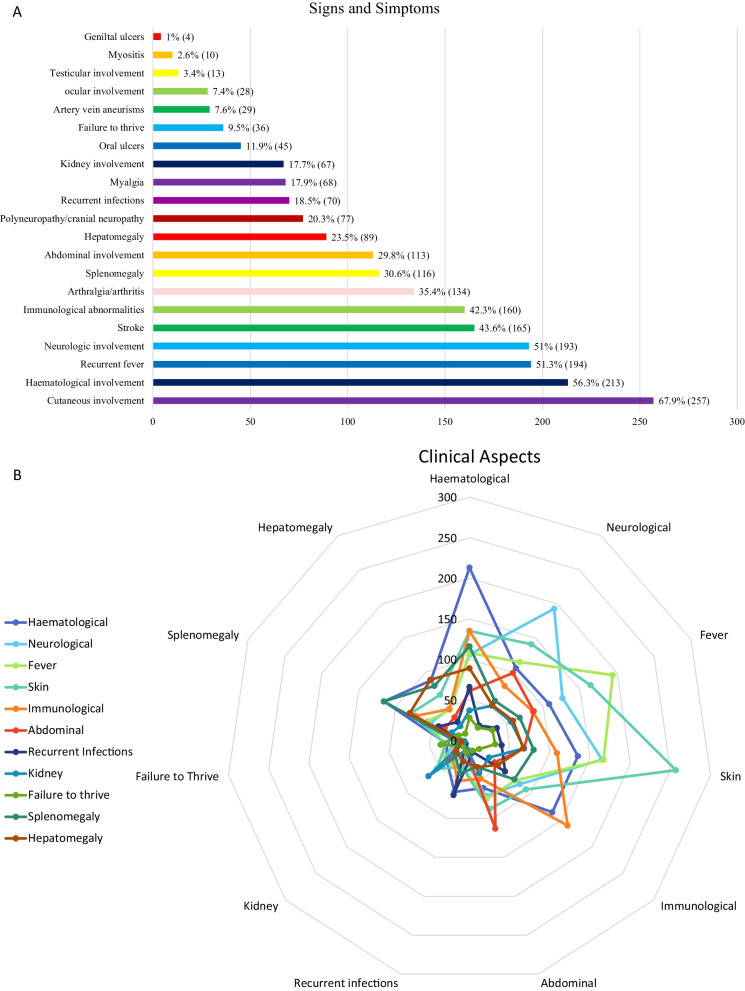
**A** shows a schematic representation of the main signs and symptoms of DADA2 patients included in this systematic review; **B** shows how the different manifestations overlap among them. Each color represents one manifestation as reported in the legend of the picture and the different points of each color the number of patients with the specific feature

The most frequent clinical characteristics described were the cutaneous (257/378, 67.9%) as livedo reticularis/racemose (180/378, 47.6%), the haematological manifestations (213/378, 56.3%), recurrent fever (194/378, 51.3%), the neurological as stroke and polyneuropathy (193/378, 51%), immunological abnormalities (160/378, 42.3%), arthralgia/arthritis (134/378, 35.4%), splenomegaly (116/378, 30.6%), abdominal involvement (113/378, 29.8%), hepatomegaly (89/378, 23.5%), recurrent infections (70/378, 18.5%), myalgia (68/378, 17.9%), kidney involvement (67/378, 17.7%), oral ulcers (45/378, 11.9%), failure to thrive (36/378, 9.5%), ocular findings (29/378, 7.6%), testicular involvement (13/378, 3.4%), myositis (10/378, 2.6%) (see Fig. 2A and B, Table 1).

**Table 1:** Main features of DADA2 syndrome

Genetic features	Mutation in ADA2: homozygous mutation or compound heterozygous or Heterozygosity
Age at onset	Widely variable Mean 92.1 months (Standard deviation 108.2)
Costitutional symptoms	Recurrent fever: not specific pattern Failure to thrive Recurrent infections
Mucocutaneous	Livedo reticularis Livedo racemose Erythema nodosum Urticarial rash Cutaneous vasculitis Leucocitoclastic vasculitis Panarteritis nodosa Skin ulcers Mouth and genital ulcers Eczema Raynaud Panniculitis Digital necrosis
Neurologic	Stroke: ischaemic and haemorrhagic Demyelinating disease Polyneuropathy, cranial neuropathy (mono/polyneuropathy) Developmental delay
Haematologic features	Anaemia (microcitic or pure red aplasia, or blackfan diamond anemia) Thrombocytopenia Lymphopenia Neutropenia Pancytopenia Hepatosplenomegaly Lymphoproliferation Lymphoma HLH
Immunological features	Predominantly humoral immunodeficiency Low B cell Low B cell switched Pan-hypogammaglobulinemia Variable NK and T cell level Increased level of Double Negative lymphocytes Interferon signature: activation of the type I IFN pathway
Lympho/splee	Splenomegaly Diffuse lymphadenopathies
Gastrointestinal	Recurrent abdominal pain with diarrhoea and vomiting Bowel ischemia/perforation/necrosis Hepatic hyper-transaminasemia Hepatomegaly
Renal	Vascular damage Haematuria
Vessel	Vasculitis: panarteritis nodosa like with the development of aneurism
Ophtalmologic	Uveitis Neuritis optica Periorbital inflammation Ocular vasculitis Papilledema Optic nerve atrophy
Musculoskeletal	Arthralgia/arthritis Myopathies
Others	Testicular involvement Recurrent warts
Treatment	IVIG and subcutaneous IG replacement → Supporting therapy Anti-TNFα → good response. (possible bridge to HSCT) HSCT → resolution of vasculitis and immunodeficiency

Among the 378 eligible patients, patients with skin manifestations were older than the others (mean 101.1 months SD ± 116.5, vs mean75.3 SD ± 88.2, p 0.041), while those with a haematological involvement (mean 64.1 months SD ± 75.6 vs mean133.1 SD ± 133.1, p < 0.001) and immunological involvement (mean 73.03 months SD ± 96.9 vs mean103.2 SD ± 112.9, p 0.05) are younger than the others. Age at the onset of the disease was not different in patients who displayed recurrent fever, neurological, ocular, abdominal, testicular and nephrological involvement.

Among the different clinical characteristics, we observed that patients with haematological manifestations showed a positive correlation with patients with immunological abnormalities (ρ = 0.417, p < 0.001), failure to thrive (ρ = 0.171, p < 0.001), recurrent infections (ρ = 0.344 p < 0.001) and a negative correlation with skin and abdominal involvement (ρ = 0.282, p < 0.001, ρ = 0.142 p 0.008 respectively) (Table 2).

**Table 2:** Table of the identified correlations among the different clinical presentations

	Haematological	Neurological	Fever	Skin	Immunological	Abdominal	Recurrent infections	Kidney	Failure to thrive	Splenomegaly	Hepatomegaly
Haematological	213	106	108	**135** **ρ. 282** **p < .001**	**135** **ρ. 417** **p < .001**	**61** **ρ. 142** **p 0.008**	**66** **ρ. 344** **p < 0.001**	37	28 **ρ. 171** **P 0.001**	**116** **ρ. 557** **P < 0.001**	**89** **ρ. 465** P < .001
Neurological	106	193	126 **ρ. 192** **p < 0.001**	**165** **ρ. 183** **p < 0.001**	**80** **ρ. 107** **P0.045**	99	**22** **ρ. 141** **p0.009**	**51** **ρ. 134** **P0.013**	19	58	52
Fever	108	**126** **ρ. 192** **p < 0.001**	194	**167** **ρ. 341** **p < 0.001**	86	**87** **ρ. 218** **p < 0.001**	37	56	**31** **ρ. 253** **P < 0.001**	68	59
Skin	**135** **ρ. 282** **p < .001**	**167** **ρ. 183** **P < 0.001**	**167** **ρ. 341** **p < 0.001**	257	109	**88** **ρ. 195** **P < 0.001**	**40** **ρ. 113** **p 0.035**	**67** **ρ. 144** **P0.007**	**32** **ρ. 125** **p0.02**	**80** **ρ. 110** **P0.041**	67
Immunological	**135** **ρ. 417** **p < .001**	82	75	92	160	41	**58** **ρ. 338** **p < 0.001**	32	16	**73** **ρ. 322** **P < 0.001**	**46** **ρ. 166** **p.003**
Abdominal	**61** **ρ. 142** **p 0.008**	**87** **ρ. 107** **P0.045**	**87** **ρ. 218** **p < 0.001**	**88** **ρ. 195** **P < 0.001**	49	113	15	**42** **P < 0.001**	13	**34** **ρ. 127** **P0.018**	34
Recurrent infections	**66** **ρ. 344** **P < 0.001**	**22** **ρ. 141** **p0.009**	33	**32** **ρ. 113** **p 0.035**	**58** **ρ. 338** **p < 0.001**	14	70	11	14 **ρ. 207** **p < 0.001**	**42** **ρ. 299** **P < 0.001**	27 **ρ. 171** **P < 0.001**
Kidney	37	**51** **ρ. 134** **P0.013**	42	**67** **ρ. 144** **P0.007**	28	**42** **ρ. 229** **P < 0.001**	6	67	**11** **ρ. 137** **p0.011**	24	21
Failure to thrive	**28** **ρ. 171** **p 0.001**	19	**31** **ρ. 253** **p < 0.001**	**32** **ρ. 125** **P0.02**	16	13	14 **ρ. 207** **p < 0.001**	**11** **ρ. 229** **p0.011**	36	15	10
Splenomegaly	**116** **ρ. 505** **p < 0.001**	53	57	**80** **ρ. 110** **P0.041**	**73** **ρ .322** **P < 0.001**	**73** **ρ. 127** **P < 0.001**	42 **ρ. 299** **P < 0.001**	24	15	116	**81** **ρ. 766** **p < 0.001**
Hepatomegaly	**89** **ρ. 465** **P < .001**	46	45	67	**46** **ρ. 160** **P0.003**	34	27 **ρ. 215** **P < 0.001**	21	10	**81** **ρ. 702** **P < 0.001**	89

Values highlighted in bold are statistically significant

Numbers into the table represent the number of children exhibiting the identified manifestations. The value of the ρ correlation has been specifically reported for each one correlation, properly adjusted for multiple correlations

p < 0.05 was considered as significant

On the other hand, patients with neurologic involvement with stroke or neuropathy showed a positive correlation with patients who showed skin manifestations (ρ = 0.183, p < 0.001), abdominal involvement (ρ = 0.142, p 0.008), recurrent fever (ρ = 0.192 p < 0.001), ocular (ρ = 0.145, p0.007), while negatively with recurrent infections (ρ = 0.141, p 0.009).

Furthermore, patients with skin manifestations showed a positive correlation with neurologic (ρ = 0.183, p < 0.001), abdominal (ρ = 0.195, p < 0.001), ocular (ρ = 0.160, p 0.003) and testicular involvement (ρ = 0.138, p 0.01), recurrent fever (ρ = 0.341, p < 0.001), failure to thrive (ρ = 0.125, p0.02),), and a negative correlation with recurrent infections (ρ = 0.113, p0.035) and haematological involvement (ρ = 0.282, p < 0.001).

While among patients with immunological abnormalities we evaluated a positive correlation with recurrent infections (ρ = 0.338, p < 0.001) and haematological alterations (ρ = 0.417, p < 0.001) and negatively with a testicular involvement (ρ = 0.107, p 0.048).

### Cutaneous manifestations

Cutaneous manifestations were reported in 257 patients (67.9%), and the mean age at onset of the disease was 101.1 months (SD ± 116.5) that is significantly higher than in the other subjects (p 0.041).

Skin manifestations, of note panarteritis nodosa-like lesions, have been reported from the first descriptions [1, 2]. Among the 257 patients with a cutaneous involvement, 180 showed a livedo reticularis or livedo racemose (47.1% of all DADA2 patients) [1, 2, 4, 9, 11, 12, 15, 16, 22, 23, 25, 26, 28–31, 34, 36, 37, 40, 43, 44, 46–48, 50, 52, 53, 56, 59, 60, 62–64, 66, 67, 69–76, 81–83, 87, 90, 91], 89 panarteritis nodosa-like lesions (23.5%) [1, 2, 5, 9, 12, 20, 21, 23, 25, 26, 28, 30, 31, 34, 35, 38, 40, 43, 44, 52, 53, 60, 64, 66, 69, 71, 80, 82–84, 87, 88, 91], 40 erythema nodosum [2, 7, 9, 11, 21, 23, 25, 28, 30, 38, 43, 44, 52, 56, 60, 63, 66, 69, 71, 82, 87, 88], 20 an urticarial rash [1, 9, 21, 22, 30, 41, 49, 61, 66, 69, 86–88], 72 an aspecific cutaneous vasculitis [1, 2, 8, 9, 11, 15, 16, 24–26, 29–31, 36, 43, 53, 61, 66, 72–75, 82, 83, 87, 91], 10 a leukocytoclastic vasculitis [2, 30, 83], 43 skin ulcers [11, 15, 21, 23, 25, 28, 30, 32, 36, 43, 53, 62, 74, 91], 30 Raynaud Phenomenon [2, 16, 21, 28, 31, 37, 43, 52, 62, 63, 69, 70, 74, 80], 39 digital necrosis [2, 8, 11, 23, 28, 31, 32, 59, 63, 66, 69, 71, 76, 81, 82], 4 eczema [21, 33, 83], 4 psoriatic lesions [30, 31, 38], 4 widespread molluscum contagiosum lesions [21, 33, 83], 4 diffuse warts cutaneous lesions [21, 33, 83]. 1 septal panniculitis [25].

### Haematological manifestations

Patients with haematological involvement, described in 213 patients (56.3%), showed a disease onset at a mean age of 64.1 months (SD ± 75.6 months).

Anaemia was reported in 97 patients (25.6%), of whom 4 showed a Blackfan-Diamond Anaemia and 23 Pure Red Aplasia [1, 2, 4, 7–9, 11, 12, 15–18, 20, 21, 29, 31, 33, 36, 40, 43, 47–49, 53, 58, 59, 62, 63, 65–67, 71, 76, 77, 82, 83, 86–89]. Thrombocytopenia was described in 34 patients (8.9%) [1, 4, 8, 9, 11, 12, 15, 17, 18, 21, 36, 40, 42, 49, 60, 65, 67, 83], neutropenia in 76 (20.1%) [1, 4, 8, 9, 11, 12, 15, 17, 18, 21, 22, 32, 36, 38, 42, 43, 45, 49, 58, 61, 63, 65, 69, 71, 77, 78, 83, 87, 90, 91], lymphopenia in 81 (21.4%) [1, 4, 5, 7–9, 11, 12, 15, 17–19, 21, 28, 32, 35, 36, 40, 42, 44, 53, 63, 65, 66, 68, 69, 71, 77–79, 81, 83, 84, 89] and pancytopenia in 18 (4.7%) [1, 4, 9, 12, 17, 18, 39, 61, 69, 83].

Lymphoproliferation was described in 21 patients (5.5%) [4, 9, 12, 15, 17, 18, 27, 36, 58, 77, 78, 83], lymphoma in 6 (1.58%) [2, 5, 9, 17, 27], of whom 3 lymphoma of Hodgkin [5, 17], leukaemia in one [9].

Splenomegaly was found in 116 patients (30.6%) [1, 4, 7–9, 12, 15–18, 22, 30, 31, 35, 36, 38–40, 58, 62–66, 68, 71, 74, 77–79, 81, 83, 85–88, 90, 91], hepatomegaly in 89 (23.5%) [1, 4, 8, 9, 12, 17, 18, 27, 28, 30, 31, 36, 49, 54, 58, 62–66, 71, 74, 78, 81, 83, 87, 90, 91], and diffuse lymphadenopathies in 27 (7.14%) [1, 4, 9, 12, 17, 18, 27, 30, 54, 58, 62, 65, 71, 86, 90].

Moreover 3 patients showed episodes of Hemophagocytic Lymphohistiocytosis [9, 28, 63].

### Immunological abnormalities

Immunological abnormalities, described in 160 patients (42.3%), were reported at a mean disease onset of 73 months (SD ± 96.9) considering the presence of immunological abnormalities with or without history of a major infection as the deficit of immunoglobulin or peculiar lymphocyte subtypes.

Among the immunological abnormalities described the most prevalent is a humoral immunodeficiency, 131 patients showed a deficit of IgM (34.6%) [1, 4, 5, 8, 9, 11, 12, 15, 21, 22, 25, 27–32, 35, 36, 39, 40, 42–44, 46, 48, 49, 53, 56, 57, 60–63, 66, 68–71, 77, 81, 83, 84, 87, 89, 90], 81 of IgG (21.4%) [1, 4, 5, 7–9, 11, 12, 15, 21, 25, 27, 30, 31, 36, 39, 42, 45, 46, 56, 57, 60, 62–64, 67–69, 71, 76, 77, 81, 83, 89, 90], 83 of IgA (21.9%) [1, 4, 5, 7–9, 12, 15, 17, 19, 21, 25, 27, 30, 31, 39, 42, 44–46, 53, 56–58, 60, 61, 63, 66, 68, 69, 71, 76, 77, 81, 83, 84, 87, 89, 90], 51 of B lymphocyte (13.5%) [1, 5, 8, 9, 12, 19, 39, 44, 45, 49, 57, 58, 61, 63, 66, 68, 71, 77–79, 83, 84, 87, 90], 19 low number switched B memory (5%) [7, 19, 21, 68, 71, 83], 23 low number of T lymphocyte (6%) [4, 5, 9, 19, 21, 27, 39, 45, 58, 61, 68, 78, 83, 84, 89, 90], and 21 low number of Natural Killer (5.5%) [8, 9, 27, 39, 45, 49, 63, 78, 83, 84, 90].

Recurrent infections were described in 70 patients (18.5%) in terms of recurrent cutaneous infections as abscesses, widespread warts or molluscum contagiosum, severe gastrointestinal infections, respiratory infections, otitis, or opportunistic infections [1, 4, 7–9, 12, 17, 19, 21, 22, 30–33, 38, 39, 42, 44, 45, 49, 58, 59, 61, 63, 64, 71, 77, 82, 83, 87, 88].

### Neurological features

Among the 378 patients, 193 (51%) patients showed a neurological involvement during the disease course. Typically, these patients showed a disease onset at mean age of 87 months (SD ± 104.3 months). Even though several papers did not report which was the first sign or symptoms in their patients, we evaluated that stroke was the first symptoms in 37 patients across the entire cohort.

The neurologic involvement associated with a humoral immunodeficiency a cutaneous involvement was the first description of this rare syndrome [1, 2].

The most frequent neurologic manifestation is the stroke that was reported in 165/378 patients (43.6%) [1, 2, 4, 5, 8, 11, 12, 15, 16, 22, 25, 26, 28–31, 34, 37, 40, 41, 43, 46, 48, 56–59, 62–66, 70, 71, 73–77, 80–82, 84, 85, 87–90]. In 146 patients showed ischaemic characteristics, while in 46 haemorrhagic. Seventy-seven patients (20.3%) showed a polyneuropathy or mononeuropathy [1, 2, 5, 11, 25, 28, 30, 31, 36, 43, 62, 63, 65, 67, 69, 74, 76, 80, 82, 85, 88].

In another 5 patients posterior reversible encephalopathy syndrome (PRES) was described [30, 45, 63, 74].

### Ocular involvement

Ocular involvement in patients with DADA2 was described in 29 patients (7.6%) in terms of uveitis in 5 patients [66, 69, 83, 87, 89], neuritis optica in 7 [23, 25, 28, 30, 63, 81], periorbital inflammation in 3 [1, 45, 67], ocular vasculitis in 12 [1, 2, 38, 59, 67, 74, 80, 89, 90], papilledema in 1 [66], optic nerve atrophy in 5 [1, 11, 67, 80, 88].

### Gastrointestinal and nephrological involvement and others

Gastrointestinal and nephrological involvement is typically secondary to vasculopathy that involved these different districts, and they were reported in 113 (29.8%) and 67 (17.7%) patients respectively.

The abdominal involvement was described in terms of recurrent abdominal pain, recurrent diarrhoea, gastrointestinal bleeding, intestinal ischaemia, bowel perforation, increased transaminase [1, 2, 4, 5, 8, 11, 12, 16, 19, 20, 22–25, 28, 30–32, 36, 37, 40, 43, 45, 47, 52, 56, 57, 59, 63–66, 69, 72, 74, 76–78, 80, 82, 87].

Nephrological involvement was characterized by renal infarct, or proteinuria, haematuria, hypertension, aneurysms of kidney arteries, glomerulonephritis [1, 2, 7, 11, 20, 23, 24, 26, 28, 31, 36, 40, 47, 52, 54, 57, 62, 63, 65, 66, 69–72, 74, 81].

Hypertension was reported in 44 patients (11.6%) [2, 20, 24, 25, 29, 40, 43, 46, 52, 54, 62, 63, 66, 73, 74, 80].

Testicular involvement in term or pain or swelling pr infarct was reported in 13 patients (3.4%) [2, 3, 24, 28, 63, 74, 91].

### Musculoskeletal symptoms

Arthralgia and arthritis were reported in 134 patients (35.44%) [2, 5, 7, 9, 12, 16, 21, 24–26, 28–31, 36, 37, 40, 43, 52, 54, 56, 60–63, 65, 66, 69, 71, 72, 74–76, 81–83, 85–87], myalgia in 58 (15.34%) [2, 5, 20, 23, 24, 28, 29, 31, 36, 41, 54, 57, 60, 62, 63, 65, 67, 69, 72, 76] and myositis in 10 (2.64%) [1, 61–63, 66, 71, 78, 84]

### Treatment and outcomes

Data about the treatment performed were available about 329/378 patients. Three hundred and seventeen (96.4%) received at least one treatment, whereas 12 (3.6%) received no therapy [9, 17, 31, 61, 74, 77]. Overall, 208 patients received an anti-TNF (63.2%), 196 corticosteroids (59.6%), 79 immunoglobulin replacement (24%), 48 cyclophosphamide (14.6%), 48 azathioprine (14.6%), 36 bone marrow transplantation (10.9%), 36 mycophenolate mofetil (10.9%), 35 methotrexate (10.6%), 31 colchicine (9.4%), 27 calcineurin inhibitors (8.2%), 20 granulocyte colony stimulating factor (GCSF) (6.1%), 19 rituximab (5.8%), 19 anticoagulant or antiaggregant treatments (5%), 17 plasma (4,5%), 13 anti-IL1 (3.4%), 9 tocilizumab (7.9%), 6 thalidomide (1.8%). Twenty-eight patients (8.5%) received other therapies including Nonsteroidal anti-inflammatory drugs (NSAIDs), analogue of prostacyclin, mesalazine, mercaptopurine, hydroxychloroquine, leflunomide, calcium channel blockers, dapsone, plasmapheresis, chemotherapy.

Among the 208 patients treated with anti-TNF alpha, just in 119, therapy outcomes have been described [1, 4, 5, 8, 9, 11, 12, 16, 17, 19–21, 23, 24, 28–32, 34–40, 43, 45, 46, 50, 53, 54, 56, 60–63, 65–67, 69, 71, 73–82, 85, 87, 89–91]. The median follow-up on anti-TNF alpha therapy was 10 months (interquartile range 6–25 months). Of these 208 patients, the data about the specific type of anti-TNF were available only in a part of patients. Fourteen were treated with adalimumab (median follow-up 8 months, IQR 6–11 months, at the dosage of 20 or 40 mg every 2 week), 54 with etanercept (median follow-up 12 months, IQR 6–24 months, dosage range 0.8–1.6 mg/kg/week), 11 infliximab (median follow-up 9 months, IQR 7.5–16.5 months, at the dose of 5 mg/kg repeated every 3–6 week). Ninety-one patients had a general improvement. Among them, 40 patients had no neurologic flares, 30 reduced the frequency of fever attacks and improved skin manifestations, 13 had improvement of gastrointestinal symptoms and 17 of musculoskeletal symptoms, 6 an improvement of haematological abnormalities, 50 showed a normalization of inflammatory index and 16 tapered or suspended the steroid therapy.

The outcome of patients who underwent bone marrow transplantation was described in 25/36 patients. Among them, 17 experienced post-transplant complications, 22 reported a complete resolution of vasculitic manifestation and haematological and immunological alterations [4, 8, 9, 12, 15, 22, 27, 43, 58, 59, 63, 77, 78]. Among the 17 patients who showed post-transplantation complications, 12 have viral reactivations or infections, 9 graft versus host diseases (of which 1 of severe grade), 4 autoimmune cytopenia (2 thrombocytopenia, 1 autoimmune haemolytic anemia, 1 neutropenia with immuno-mediated pure red cell aplasia), 1 ileo-ileal intussusception, 1 bronchiectasis, 1 refractory severe thrombocytopenia, 1 poor B cell function.

A general improvement was reported also in patients who received steroids (20 cases), thalidomide (6), cyclosporine (3 patients, in 2 cases with corticosteroids), mycophenolate mofetil (5 patients, in 1 case associated to corticosteroids), azathioprine (2), tocilizumab (1), anti-IL-1 (1).

In total, 32 patients died (8.4%). Six patients deceased from infectious disease, 5 from stroke, 4 from intestinal perforation, 2 from causes not related to the disease, 1 from pulmonary haemorrhage, 1 from hypovolemic shock; in 13 cases the cause of exitus was not clarified [1, 2, 5, 7, 8, 11, 15, 20, 28, 31, 32, 38, 41, 43, 63, 69, 74, 77, 78].

## Discussion

Since the first two independent reports were published [1, 2], the knowledge about this rare syndrome have been expanded in terms of disease presentation and treatment availability. Paediatricians and adult physicians should be aware of the main features of this disease in order to promptly achieve diagnosis as well as prevent severe complications and improve quality of life.

Our systematic review summarized the 378 reported cases so far, including the spectrum of phenotypic manifestations, therapeutic approaches, and clinical outcomes in this rare syndrome.

As monogenic auto-inflammatory disease, although it onset mainly in childhood, clinical signs and symptoms of DADA2 syndrome may also start in adulthood. Up to the 8.5% of patients may have the first signs or symptoms after 18 years old, and the 25.4% after 10 years old. Taking into account the quite recent description of the disease, it is not surprising that the diagnosis may has been performed over the adult age. Additionally, some of the patients with adult onset might have had scares and/or moderate signs over the childhood that had been misdiagnosed. However, the wide age distribution and the incomplete penetrance seems to be a common feature to the autoinflammatory diseases.

We address that the clinical presentation of DADA2 syndrome is polyhedric, showing a spectrum of manifestations, ranging from the panarteritis nodosa-like lesions to the humoral immunodeficiency, recurrent fever, and stroke. Haematological abnormalities, ocular findings, inflammatory bowel diseases are just some of the clinical signs of the expanding phenotype of the DADA2 deficiency. Of note, paediatric and adult neurologist should consider that stroke might be the first and isolated sign of the disease as demonstrated in several patients.

Compared to single case observations, the aggregate data of this systematic review showed an interesting difference according to the age of onset. In early childhood, haematological and immunological manifestations result predominant, while skin and vasculopathy manifestations show a later onset. These differences might be the result of a different residual enzymatic activity, thus exhibiting different manifestation over different age of life.

However, a specific genotype–phenotype correlation cannot be excluded. In aall the reported cases a biallelic pathogenetic, or likely pathogenetic mutation has been identified into *ADA2* gene. Two cases are the exception: a discernible heterozygous mutation in one, and an unknown mutation in the other one [62]. Nonetheless this confounding genetic results, the two patients were anyway affected since they showed an abnormal ADA2 activity [62]. Currently, the pathogenetic and/or likely pathogenetic mutations are located over the entire coding region of gene and a selective a preferential location for each phenotype has not been identified.

Over the years, several attempts have been performed in order to point out a genotype phenotype correlation, but according several expert opinion, patients of same family with the same genotype (same biallelic mutations) show anyway different phenotype, with a width range of severity [8, 9, 92, 93]. It has been hypothesized (Lee PY) that mutations able to significantly affect the enzymatic function lead to a more severe phenotype, such as the stroke and/or hematologic involvement. However, due to an incomplete penetrance and the high prevalence of compound heterozygosity, a precise genotype–phenotype correlation model cannot be predicted [8, 9].

A scarce or null enzyme activity has been detected in patients with pure red cell aplasia or severe stroke or overall, with severe phenotype, as well as an higher enzyme activity has been reported in patients with really mild phenotype or isolated cutaneous involvement [8].

The type of mutation, the key role in determining the severity of the phenotype seems associated to the amount of residual enzymatic activity: greater is the residual availability of the enzyme, lower is the severity of the phenotype. Otherwise, it not properly known what decreased production of ADA2 metabolites means. Most of the diagnostic studies measures ADA2 activity in vitro, rather than a direct measurement of adenosine or inosine concentration [8, 30].

In order to improve timing diagnosis and management planning, different specialists, including pediatrician, neurologists, hematologists, rheumatologists, immunologists, internal medicine specialists, ophthalmologists and nephrologists should increase knowledge and awareness of this disease. In this clinical setting a close multidisciplinary approach might be settled up. Specific clinical situations as early onset stroke, immunologic alterations associated to cutaneous vasculitis might probably require systematic determination of DADA2 enzyme evaluation to timely discover the disease and timely prescribe appropriate treatment and follow-up.

Furthermore, the increased knowledge regarding pathophysiology of DADA2 deficit opened the way to new possible treatments. TNFα has been evaluated as key cytokine in the onset of the vasculopathy manifestations. Currently, a tailored treatment with anti-TNFα agents shows a crucial role in treating patients with a vasculopathy phenotype [1, 4, 5, 8, 9, 11, 12, 16, 17, 19–21, 23, 24, 28–32, 34–40, 43, 45, 46, 50, 53, 54, 56, 60–63, 65–67, 69, 71, 73–82, 85, 87, 89–91]. Bone marrow transplantation currently represent a real opportunity for those patients where the hematological involvement is prevalent [4, 8, 9, 12, 15, 22, 27, 43, 58, 59, 63, 77, 78], even though some complications were reported. In recent studies, which have evaluated in deep the physio pathological mechanism underlined this rare syndrome, was highlighted a positive Interferon-signature that might suggest a possible role for JAK-inhibitors [50, 62, 75, 93]. Moreover, Zoccolillo et al. [94] in a recent elegant study demonstrated a successful treatment with early gene therapy to correct ADA2 activity. However, at the moment a gene therapy for DADA2 is not available, although research is ongoing.

Before drawing firm conclusions, caveats and limitations of our systematic review need to be considered and discussed.

We included single case reports to avoid missing any peculiar phenotype of such a rare disease. We recognize that this may led to a positive selection bias (publication bias); the rarity of the disease might also overcome this issue. Additionally, the different nature of the selected papers (registries, prospective studies, retrospective studies, case series, case reports), and the different aims of each one of them may hamper the possibility to aggregate extracted data from different study designs. However, since a genetic confirmatory diagnosis for DADA2 has been used as selection criteria of the index population, the cumulative count of different clinical manifestations is referred to an homogenous population regardless the study type. Since the different study design and aims of the eligible studies included, some clinical manifestations might be under-reported even though the patients showed additional clinical manifestations.

A consistent correlation between age at onset/clinical manifestations and ADA2 enzymatic could not be performed since this datum were not extractable from most of the papers that survived the inclusion/exclusion selection criteria of this systematic review.

A duplicated case bias cannot be excluded. As reported in methodology section, we tried to avoid this bias following the PRISMA guidelines advisees for this issue. However, if f any doubt, we adopted a conservative method excluding the index patient suspected to be a bias.

Eventually, taking into account the overall low prevalence of DADA2 syndrome, the small number of available studies and participants is not completely unexpected and seems to represent the real situation in clinical practice. Notably, we did not include studies when it was not possible to extract information on potentially eligible patients.

## Conclusion

Due to this highly variable phenotype and age of presentation, patients with DADA2 may present to several type of specialists. Given the important morbidity and mortality, early diagnosis and treatment are mandatory. TNF-inhibition is successful in preventing vascular events, while hematopoietic cell transplantation is an option in case of hematological disease and immunodeficiency. ADA2 protein, gene therapy or Jak-inhibitors might be promising future therapies, but additional studies on the ADA2 deficiency pathophysiology are necessary.

## Supplementary Information

**Additional file 1**. **Table S1**: ADA2 variants identified from literature review.

## Data Availability

The datasets used and/or analysed during the current study are available from the corresponding author on reasonable request.

## Abbreviations

DADA2: Deficiency of adenosine deaminase 2
PAN: Polyarteritis nodosa
CECR1: Cat eye syndrome chromosome region candidate 1
TNF-α: Tumor necrosis factor-alpha
HSCT: Hematopoietic stem cell transplantation
