# Supplementary material for: A wide spectrum of phenotype of deficiency of deaminase 2 (DADA2): a systematic literature review
Source: Orphanet J Rare Dis. 2023 May 13;18:117. doi: 10.1186/s13023-023-02721-6 (PMC10183141; doi:10.1186/s13023-023-02721-6)
Supplement: Supplementary file 1 — Additional file 1. Table S1: ADA2 variants identified from literature review. [file 13023_2023_2721_MOESM1_ESM.docx]

Table 1: **ADA2 variants identified from literature review.**

| Mutation | Domain |
| --- | --- |
| c.-47+2T>C | Intron 1 |
| c.37_39del, p.Lys13* | Mutation in trans |
| c.2T>C, p.Met1Thr | Exon 2 |
| c.25C>T, p.Arg9Trp | Exon 2 |
| c.73G>T, p.Gly25Cys | Exon 2 |
| c.-2484del, c.114delC | 5 Flanking |
| c.133C>T, p.Arg45Trp | Exon 2 |
| c.140G>C, p.Gly47Ala | Exon 2 |
| c.139G>A, p.Gly47Arg | Exon 2 |
| c.140G>T, p.Gly47Val | Exon 2 |
| c.139G>T, p.Gly47Trp | Exon 2 |
| c.142G>A, p.Gly48Arg | Exon 2 |
| c.143dup, p.Arg49Alafs*13 | Exon 2 |
| c.144delG, p.Arg49Glyfs*4 | Exon 2 |
| c.144dupG, p.His91Alafs*4 | Exon 2 |
| c.232_322+105delinsATG | Exon4 |
| c.272A>G, p.His91Arg | Exon2 |
| c.278T>C, p.Ile93Thr | Exon2 |
| c.326C>A, p.Ala109Asp | Exon 3 |
| c.336C>G, p.His112Gln | Exon3 |
| c.334C>T, p.His112Tyr | Exon 3 |
| c.396_397del p.His133Leufs*44 | Exon 3 |
| c.476G>A, p.Cys159Tyr | Exon 3 |
| c.427del, p.Ile143Serfs*41 | Exon 3 |
| c.505C>G, p.Arg169Gly | Exon 3 |
| c.506G>A, p.Arg169Gln | Exon 3 |
| c.542+1G>A | Intron 3 |
| c.533T>C, p.Phe178Ser | Exon 3 |
| c.562C>G, p.Leu188Val | Exon4 |
| c.563T>C, p.Leu188Pro | Exon 4 |
| c.578C>T, p.Pro193Leu | Exon 4 |
| c.620T>C, p.Phe207Ser | Exon 4 |
| c.629delT, p.Ile210Thrfs*57 | Exon 4 |
| c.634_636delTTC, p.Phe212del | Exon 4 |
| c.650T>A, p.Val217Asp | Exon4 |
| c.660C>A, p.Tyr220* | Exon 4 |
| c.680_681delAT, p.Tyr227Cysfs*27 | Exon 4 |
| c.706_708del, p.Tyr236del | Exon 4 |
| c.709del, p.Glu237Argfs*30 | Exon 4 |
| c.712G>A, p.Asp238Asn | Exon 4 |
| c.744delG, p.Arg248Serfs*19 | Exon 4 |
| c.728T>G, p.Met243Arg | Exon 4 |
| c.746T>C, p.Leu249Pro | Exon 4 |
| c.752C>T, p.Pro251Leu | Exon 4 |
| c.753G>A, p.Pro251Pro | Exon 4 |
| c.753+168_754-229_1081+139_1082-92del, p.(Val252Thrfs*7) | Exon 4 |
| 753+2T>A | Intron 4 |
| c.791G>C, p.Trp264Ser | Exon 5 |
| c.794C>G, p.Ser265* | Exon 5 |
| c.1190A>G, p.Tyr397Cys | Exon 5 |
| c.872C>T, p.Ser291Leu | Exon 5 |
| c.916C>T, p.Arg306* | Exon 6 |
| c.927G>A, p.Met309Ile | Exon 6 |
| c.934C>T, p.Arg312* | Exon 6 |
| c.962G>A, p.Gly321Glu | Exon 6 |
| c.962G>C, p.Gly321Ala | Exon 6 |
| c.972+3 A>G | Intron 6 |
| c.973 -2A>G | Intron 6 |
| c.973- 1G>A | Intron 6 |
| c.982G>A, p.Glu328Lys | Exon 7 |
| c.1159C>A, p.Leu387Met | Exon 7 |
| c.1031C>T, p.Pro344Leu | Exon 7 |
| c.1052T>A, p.Leu351Gln | Exon 7 |
| c.1069G>A, p.Ala357Thr | Exon 7 |
| c.1057T>C, p.Tyr353His | Exon 7 |
| c.1065C>A, p.Phe355Leu | Exon 7 |
| c.1114 G>A, p.Val372Met | Exon 7 |
| c.1069G>A, p.Ala357Thr | Exon 7 |
| c.1072G>A, p.Gly358Arg | Exon 7 |
| c.1078A>G, p.Thr360Ala | Exon 7 |
| c.1110C>A, p.Asn370Lys | Exon 8 |
| c.1147G>A, p.Gly383Ser | Exon 8 |
| c.1148G>A, p.Gly383Asp | Exon 8 |
| c.1196G>A, p.Trp399* | Exon 8 |
| c.1211T>C, p.Phe404Ser | Exon 8 |
| c.1223G>A, p.Cys408Tyr | Exon 8 |
| c.1225C>T, p.Pro409Ser | Exon 8 |
| c.1269C>G, p.Asn423Lys | Exon 9 |
| c.1337T>C, p.Phe446Ser | Exon 9 |
| c.1348G>T, p.Gly450Cys | Exon 9 |
| c.1346_1347insTT, p.Lys449Asnfs*2 | Exon 9 |
| c.1352T>G, p.Leu451Trp | Exon 9 |
| c.1358A>G, p.Tyr453Cys | Exon 9 |
| c.1360G>C, p.Asp454His | Exon 9 |
| c.1367A>G, p.Tyr456Cys | Exon 9 |
| c.1373T>A, p.Val458Asp | Exon 9 |
| c.1392dup, p.Met465Aspfs*4 | Exon 9 |
| c.1397_1403delAGGCTGA, p.Lys466Thrfs*2 | Exon 9 |
| c.1445A>G, p.Tyr482Cys | Exon 10 |
| [c.1501T>A](https://varsome.com/variant/hg38/ADA2(ENST00000399837.8):c.1501T%3EA?&annotation-mode=germline) p.Trp501Arg | Exon 10 |
| c.882-2A>G | Intron 5 |
| c.962G>A, p.Gly321Glu | Exon 6 |
| c.972+359_973-153_1081+149_1082-200del, p.Val325Thrfs*7 | Exon 7 |
| c.1240_1442del (deletion of exon 9) | Exon 9 |
| c.1447_1451Del, p.Ser483Profs*5 | Exon 10 |
